# P_VggNet: A convolutional neural network (CNN) with pixel-based attention map

**DOI:** 10.1371/journal.pone.0208497

**Published:** 2018-12-12

**Authors:** Kunhua Liu, Peisi Zhong, Yi Zheng, Kaige Yang, Mei Liu

**Affiliations:** Advanced Manufacturing Technology Center, Shandong University of Science and Technology, Qingdao, Shandong province, China; Newcastle University, UNITED KINGDOM

## Abstract

Attention maps have been fused in the VggNet structure (EAC-Net) [1] and have shown significant improvement compared to that of the VggNet structure. However, in [1], E-Net was designed based on the facial action unit (AU) center and for facial AU detection only. Thus, for the use of attention maps in every image type, this paper proposed a new convolutional neural network (CNN) structure, P_VggNet, comprising the following parts: P_Net and VggNet with 16 layers (VggNet-16). The generation approach of P_Net was designed, and the P_VggNet structure was proposed. To prove the efficiency of P_VggNet, we designed two experiments, which indicated that P_VggNet could more efficiently extract image features than VggNet-16.

## 1 Introduction

Normally, the traditional image recognition process can be divided into the following steps: image collection, image feature extraction, and image recognition and classification (e.g., local binary pattern (LBP) + support vector machines (SVM) [2–3], histogram of oriented gradients (HOG) + SVM [4–5]). However, deep learning-based approaches (for example, convolutional neural networks (CNNs [6–10]) and stacked autoencoder (SAE) [11–12]) have been proven to be efficient approaches for image recognition and have been widely used. Deep learning-based approaches are popular mainly because they can efficiently learn deep features better than traditional image feature extraction methods can. Therefore, deep learning-based approaches can improve accuracy.

To further improve the accuracy of CNNs, we propose a new CNN structure that can more efficiently learn deep image features, P_VggNet. P_VggNet comprises the following parts: P_Net and VggNet with 16 layers (VggNet-16). VggNet-16 is the basic framework of P_VggNet, and P_Net was added to VggNet-16 to create an enhanced net. To validate P_VggNet, we compared it to VggNet-16 with two experiments. Experiment 1 was trained and tested on two different datasets (the Canadian Institute for Advanced Research (CIFAR)-10 and CIFAR-100 datasets) for image recognition. This experiment indicated that regardless of the use of the CIFAR-10 or CIFAR-100 dataset, P_VggNet can obtain higher accuracy and lower loss than VggNet-16. Experiment 2 was designed to test the face detection performance of the CNNs. We changed the convolutional layers in ONet of Multitask Cascaded Convolutional Neural Networks (MTCNN) to P_VggNet and VggNet-16, and trained the two MTCNN models on the Web Image Dataset for Event Recognition (WIDER) Face dataset and Celeba dataset. The results from the Labeled Wikipedia Faces (LWF) dataset and Face Detection Dataset and Benchmark (FDDB) dataset demonstrated that the true positive rate of modeling with P_VggNet is higher than that with VggNet-16.

The remainder of this paper is organized as follows. The related work is introduced in section 2. The generation approach of P_Net is presented in section 3. The P_VggNet architecture is provided in section 4. The P_VggNet structure and VggNet-16 structure experiments are discussed in section 5. Finally, section 6 summarizes this paper.

## 2 Related work

Many classic CNNs for image classification have been proposed and have achieved remarkable results. Some examples are as follows. LeCun et al. [13] proposed LeNet-5 for document recognition; LeNet-5 comprised four convolutional layers and two fully connected layers. In 2012, Krizhevsky et al. [14] proposed Alexnet, which was composed of five convolutional layers and two fully connected layers, and it achieved a 16.4% error rate on ImageNet Large Scale Visual Recognition Challenge (ILSVRC) 2012. In 2014, Simonyan et al. [15] proposed VggNet, which added more convolutional layers than Alexnet did. Reference [15] indicated that the top-5 error rate of VggNet-16 in ILSVRC 2013 was 7.3%, and for VggNet-19, the top-5 error rate decreased to 6.8%. In the same year, Szegedy et al. [16] invented GoogleNet, which has a total of 34 levels of convolutional layers and fully connected layers. By adopting the inception module and carefully increasing the depth and width of the network, GoogleNet decreased the top-5 error rate of ILSVRC 2014 to 6.7%. In 2015, He et al. [17] found that at some level, with an increase in CNN layers, CNNs can obtain better accuracy. However, with the addition of more layers to CNN layers, the error rate of CNNs increased. Therefore, He et al. adopted residual representations and shortcut connections, thereby providing ResNet. The error rate of ResNet-152 on the ImageNet 2012 classification dataset was 4.49% and was the first CNN to surpass the reported human-level performance (5.1%, [18]) on ILSVRC 2014. With the depth increase in CNNs, the accuracy increased. In addition, the improvement in the CNNs mostly focused on the depth of CNN structures. Usually, deeper CNN structures result in higher CNN accuracy.

The positive association between CNN structure depth and accuracy is due to the extraction of more valuable features by deeper layers of CNN structure. However, as the depth of CNN structures increases, the model data and training time also increase. These findings leading to the question of whether valuable features can be more efficiently extracted with fewer convolutional layers. Residual representations and shortcut connections [17] represent a proven efficient approach for extracting features. In addition, attention maps are another efficient approach for extracting features. An attention layer [19] has been used to identify interesting areas to provide better answers in a visual question-answering task and achieved remarkable results. Moreover, a salient map (or attention map) [20] has been used to describe the important subareas of an image. Li et al. [1] added enhancing layers to VggNet (E-Net, with an attention map) to extract more valuable features. However, E-Net is designed based on facial action unit (AU) centers, which can extract facial features only. For other types of images (i.e., plane, train, car, and house), E-Net is useless.

To overcome the drawbacks of E-Net, we proposed a new E-Net generation approach, P_Net. P_Net is an array of weights that is based on the pixels of every image. Higher pixels correspond to larger weights, and lower pixels correspond to smaller weights. Then, we added P_Net to VggNet-16 to generate a new structure, P_VggNet. The experiments on the CIFAR-10 and CIFAR-100 datasets indicated that P_VggNet can better learn deep features and achieve higher accuracy and lower loss than VggNet can. The experiments on the LWF dataset and FDDB dataset demonstrated that the true positive rate of modeling with P_VggNet is higher than that with VggNet-16.

## 3 P_Net

P_Net was inspired by E_Net in the literature [1]. In [1], Li et al generated E_Net by the following three steps:

1. Obtained the landmarks for the key points on the face and facial AU centers by shifting a distance or directly using existing facial landmarks.
2. Generated the facial attention map. For each AU center, a higher weight is assigned to closer points to the AU center. The relationship follows the equation:
  $$w=1-0.095d_{m}$$ (1)
  where *d*_*m*_ is the Manhattan distance to the AU center.
3. Added the facial attention map as enhancing layers to group 3 and group 4 of the VggNet structure. The enhancing layers were called E_Net.

The core of E_Net is the generated facial attention map. Therefore, E_Net adapts to face images only. There is no AU center or facial attention map for other types of images.

**Fig 1:**
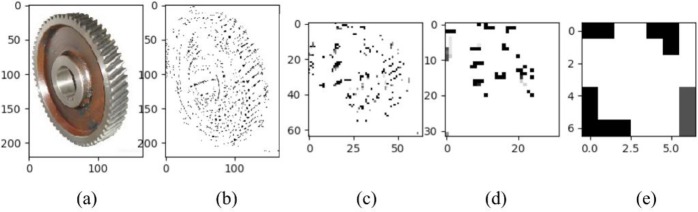
The original image of a gear and its feature map of sizes 220×165, 64×64, 32×32, and 7×7.

To generate the attention map for all types of images, we propose the generation of the attention map by the pixels of images. LBP [21] is one classical feature extraction approach that changes image pixel values to weight values. If a pixel is larger than one pixel number, which is set by the researchers, then LBP sets the weight of this pixel to 1. Otherwise, the weight is 0. We experimented with LBP on a gear image with different sizes (220×165, 64×64, 32×32, 7×7). In this experiment, the parameters of LBP are set as follows: the radius is 3, and the number of points is 8. Fig 1(A), 1(B), 1(C), 1(D) and 1(E) shows the original image of a gear and its feature map of sizes 220×165, 64×64, 32×32, and 7×7. As shown, LBP can extract image features if the image has many pixels (Fig 1(B)). However, for images with few pixels, LBP can barely extract image features (Fig 1(E)).

We tested images with few pixels because the attention map will be added after group 3 and group 4 of VggNet-16 structure, and the attention map size should match the feature map size of group 3 and group 4. However, as the pooling layers increase, the feature map size of group 3 and group 4 are much smaller than the original image. Therefore, the attention map generated by LBP is inefficient.

To ensure that the generated attention map can efficiently extract image features, we propose an attention map generation approach, the pixel-based attention map. In this approach, we set the weight according to the pixel value. The weight is divided into 10 grades, and the range of the weight value is (0–1). Higher weights are assigned to larger pixels. The relationship between weight and pixel is shown in Table 1.

**Table 1:** The relationship between weight and pixel.

Pixels	Weight
0~26	0.1
27~52	0.2
53~78	0.3
79~104	0.4
105~130	0.5
131~156	0.6
157~182	0.7
183~208	0.8
209~234	0.9
235~255	1

According to Table 1, the attention map of Fig 1(A) in size 7×7 is:

$$\left[\begin{array}{ccccccc} 1.0 & 0.9 & 0.3 & 0.2 & 1.0 & 1.0 & 1.0\\ 0.5 & 0.4 & 0.4 & 0.5 & 0.3 & 1.0 & 1.0\\ 0.5 & 0.4 & 0.4 & 0.2 & 0.6 & 0.4 & 1.0\\ 0.6 & 0.6 & 1.0 & 0.6 & 0.4 & 0.4 & 1.0\\ 1.0 & 0.1 & 0.5 & 0.3 & 0.3 & 0.5 & 1.0\\ 1.0 & 0.6 & 0.2 & 0.3 & 0.4 & 0.5 & 1.0\\ 1.0 & 1.0 & 1.0 & 0.4 & 1.0 & 1.0 & 1.0 \end{array}\right]$$

**Fig 2:**
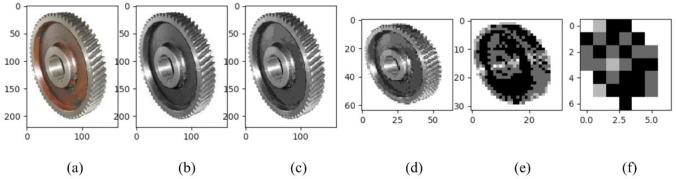
The original image of a gear, its grayscale image, and its feature map of sizes 220×165, 64×64, 32×32, and 7×7.

To visualize the attention map, we magnify the weight by 100 times. The results are shown in Fig 2. Fig 2(A), 2(B), 2(C), 2(D), 2(E) and 2(F) are the original image of a gear, its grayscale image, and its feature map of sizes 220×165, 64×64, 32×32, and 7×7, respectively. Fig 2 shows more image features than does Fig 1, indicating that our proposed attention map generation approach is more efficient than LBP.

**Fig 3:**
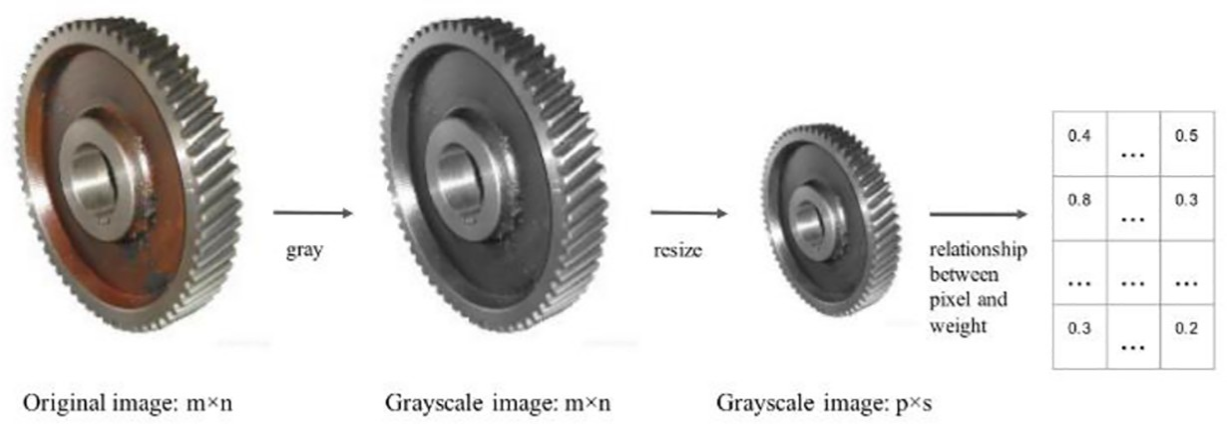
The pixel-based attention map generation approach.

In consideration of the previous image processing, the pixel-based attention map approach can be generated by the following steps (Fig 3):

Step 1: Convert the original images to grayscale images.
Step 2: Resize the grayscale images. Because the attention map will be added to VggNet, the size of the grayscale images should equal to the corresponding feature map size (an example of the grayscale image size is shown in section 5).
Step 3: Generate the attention map according to the relationship between weight and pixel (Table 1).

We added the pixel-based attention map as enhancing layers to group 3 and group 4 of the VggNet structure. The enhancing layers are called P_Net.

## 4 P_VggNet structure

**Fig 4:**
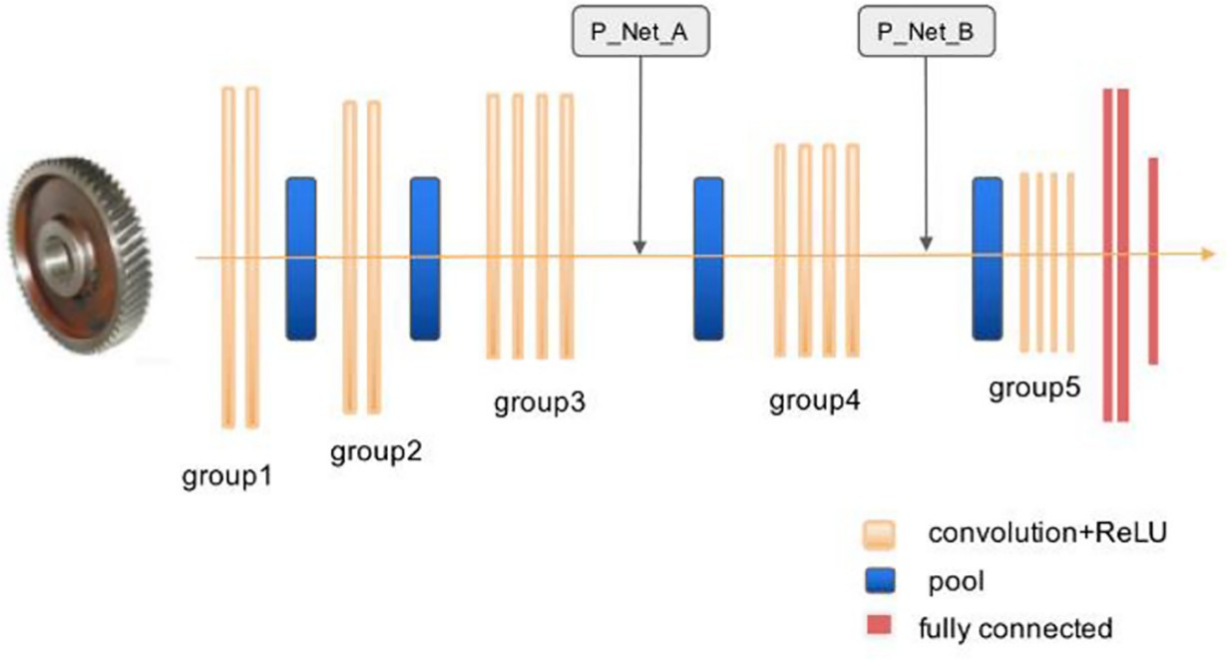
P_VggNet structure.

The P_VggNet structure (Fig 4) consists of the following parts: P_Nets and VggNet-16. We chose the 16-level VggNet as the basic network architecture and P_Nets as a residual part added into VggNet. We added two P_Nets after group 3 and group 4, and the two corresponding P_Nets are called P_Net_A and P_Net_B. Because P_Nets were added after group 3 and group 4, when we generated P_Net, the size of P_Net_A equaled the feature map size of group 3. In addition, the size of P_Net_B equaled the feature map size of group 4.

Additionally, in the classic VggNet-16 structure,

Pool3=MaxPool(Conv3_3) (2)

Pool4=MaxPool(Conv4_3) (3)

where “Conv m_n” is the convolutional layer output in the n-th layer of the m-th group, and “Pool m” is the output of the m-th group.

However, P_VggNet is VggNet-16 with P_Nets, and the forward calculation formula between P_Net_A and P_Net_B is also different. To derive this forward calculation formula, we defined the related parameters as follows:

Input_A: the input of Pool 3;

Input_B: the input of Conv5_1;

Thus, after P_Net_A, the input of P_VggNet can be described as

Input_A=Conv3_3+P_Net_A (4)

Then,

Pool3=MaxPool(Input_A) (5)

Likewise,

Input_B=Conv4_3+P_Net_B (6)

Pool4=MaxPool(Input_B) (7)

The other forward calculation formulas of P_VggNet are the same as those in VggNet. In addition, in P_VggNet, the activation function is a rectified linear unit (ReLU) [22], and the loss function is a cross-entropy cost function. The pooling method after convolutional layers is max pooling, and the pooling method after fully connected layers (fc 1 and fc 2) is average pooling.

## 5 Experiments

To prove the correctness and efficiency of the P_VggNet structure, we compared P_VggNet to VggNet-16. We designed two experiments to test the performance on image recognition and face detection.

1) Experiment 1

Experiment 1 was trained and valuated on the CIFAR-10 dataset [23] and CIFAR-100 dataset [23]. When training P_VggNet and VggNet, we randomly separated the data into the following parts: 70% data for training, 20% data for testing and 10% data for valuation. Because the data sizes of the CIFAR-10 dataset and CIFAR-100 dataset are all 32×32, the parameters of P_VggNet and VggNet were set to approximately the same values. The concrete parameter settings are shown in Table 2.

**Table 2:** Parameter settings.

Input	Parameter number	Kernel	Feature map	Stride
Image	3072		32×32	
Conv 1_	65536	[3×3, 64], 2	32×32	1
Poo l	16384	[2×2], 1	16×16	2
Conv 2_	32768	[3×3, 128], 2	16×16	1
Poo 2	8192	[2×2], 1	8×8	2
Conv 3_	16384	[3×3, 256], 3	8×8	1
Poo 3	4096	[2×2], 1	4×4	2
Conv 4_	8192	[3×3, 512], 3	4×4	1
Poo 4	2048	[2×2], 1	2×2	2
Conv 5_	2048	[3×3, 512], 3	2×2	1
Poo 5	512	[2×2], 1	1×1	2
Fc 1	4096	Average pool	1×1	
Fc 2	4096	Average pool	1×1	
Fc 3	10			

In the P_VggNet structure, P_Net_A and P_Net_B were added to group 3 and group 4, respectively. As seen from Table 2, the feature maps of group 3 and group 4 are 8×8 and 4×4, respectively. Therefore, the resized P_Net_A and P_Net_B should be 8×8 and 4×4, respectively. In addition, when training, the batch size is 128, and the maximum number of steps is 8000.

(1) Experiment on the CIFAR-10 dataset

**Fig 5:**
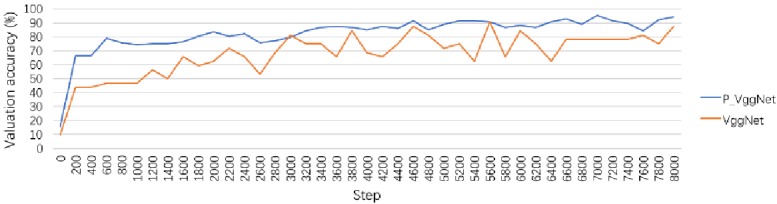
Validation accuracy curve of P_VggNet and VggNet.

We first trained P_VggNet and VggNet on the CIFAR-10 dataset. Fig 5 shows the valuation accuracy of two structures. As seen in Fig 5, the accuracy of both structures increased rapidly during the initial training. However, overall, the accuracy curve of P_VggNet always exceeded that of VggNet and was more stable than that of VggNet. After 8000 steps, the valuation accuracy of P_VggNet is 94.2%, and the valuation accuracy of VggNet is 87.5%.

**Fig 6:**
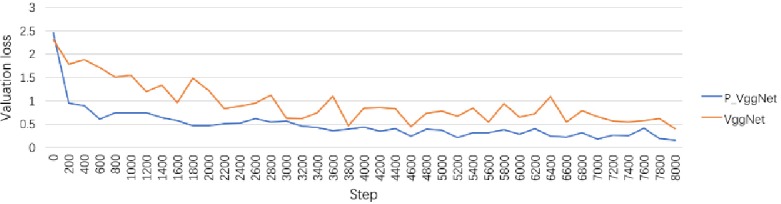
Validation loss curve of P_VggNet and VggNet.

Fig 6 is the corresponding valuation loss curve of P_VggNet and VggNet. Similar to the valuation accuracy curve, the loss of both structures decreased rapidly during the initial training. However, overall, the loss curve of P_VggNet was always lower than that of VggNet, and P_VggNet was more stable than VggNet was. After 8000 steps, the valuation loss of P_VggNet is 0.26, and the valuation loss of VggNet is 0.41.

We tested P_VggNet and VggNet on 9984 images; the average accuracy of P_VggNet and VggNet is 88.90%, and 77.15%, respectively. In addition, the training time of the two structures was monitored. With the OS X El Capitan system, Intel Core i5, and TensorFlow 1.2.1 (CPU), the use of VggNet on the CIFAR-10 dataset took approximately 14 hours. However, P_VggNet took approximately 16 hours.

(2) Experiment on the CIFAR-100 dataset

**Fig 7:**
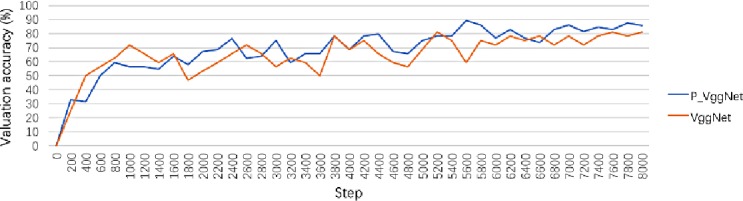
Validation accuracy curve of P_VggNet and VggNet.

In this experiment, P_VggNet and VggNet were trained on the CIFAR-100 dataset. The valuation accuracy curve is shown in Fig 7. As seen in Fig 7, after 8000 steps, the valuation accuracy of P_VggNet is 85.93%, and the valuation accuracy of VggNet is 81.25%. The highest accuracy values of both P_VggNet and VggNet occurred at step 5600 and are 89.06% and 81.25%, respectively.

**Fig 8:**
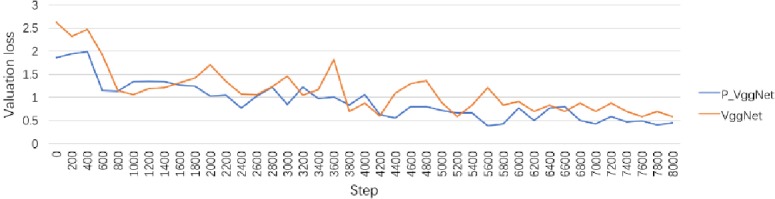
Validation loss curve of P_VggNet and VggNet.

The valuation accuracy curve is shown in Fig 8. As seen in Fig 8, after 8000 steps, the valuation loss of P_VggNet is 0.45, and the valuation accuracy of VggNet is 0.72. The lowest accuracy values of both P_VggNet and VggNet occurred at step 5600 and are 0.38 and 0.57, respectively.

We tested P_VggNet and VggNet on 9984 images, and the average accuracy of P_VggNet and VggNet is 79.20%, and 74.73%, respectively. With regard to the training time, we chose the same equipment for this equipment as that of the above experiment. The VggNet-16 structure took approximately 21 hours on the CIFAR-100 dataset, and P_VggNet took approximately 24 hours.

In summary, regardless of whether we used the CIFAR-10 dataset or the CIFAR-100 dataset, the following occurred: (1) Compared to the CIFAR-100 dataset, the CIFAR-10 dataset is easier to train, and it can obtain better accuracy and loss. (2) The valuation accuracy of P_VggNet is higher than that of VggNet-16, and the valuation loss of P_VggNet is lower than that of VggNet-16. Additionally, P_VggNet required more training time than did VggNet.

2) Experiment 2

MTCNN [24] is an efficient framework for face detection and alignment and includes the following stages of deep convolutional networks: PNet, RNet and ONet. To prove that P_VggNet is more efficient than VggNet, we simply changed the convolutional layers in ONet to P_VggNet and VggNet. Both models were trained on the WIDER Face dataset [25] and Celeba dataset [26]. The WIDER Face dataset is for face detection, and the Celeba dataset is for landmark detection. Because the input image size of ONet is 48×48, the parameter setting of ONet with P_VggNet or VggNet is shown in Table 3. Therefore, the size of P_Net_A and P_Net_B is 12×12 and 6×6, respectively.

**Table 3:** Parameter settings of ONet.

Input	Parameter number	Kernel	Feature map	Stride
Image	6,912		48×48	
Conv 1_	147,456	[3×3, 64], 2	48×48	1
Poo l	36,864	[2×2], 1	24×24	2
Conv 2_	73,728	[3×3, 128], 2	24×24	1
Poo 2	221,184	[2×2], 1	12×12	2
Conv 3_	36,864	[3×3, 256], 3	12×12	1
Poo 3	9,216	[2×2], 1	6×6	2
Conv 4_	18,432	[3×3, 512], 3	6×6	1
Poo 4	4,608	[2×2], 1	3×3	2
Conv 5_	4,608	[3×3, 512], 3	3×3	1
Poo 5	512	[3×3], 1	1×1	2
Fc 1	4096	Average pool	1×1	
Fc 2	4096	Average pool	1×1	
Fc 3	10			

For parameters, the activation function is ReLU, and the optimization algorithm is stochastic gradient descent (SGD). The other parameters follow the parameters given by reference [24]. The two models were trained on Ubuntu 16.04, TensorFlow 1.2.1, and three Tesla k40c GPU.

**Fig 9:**
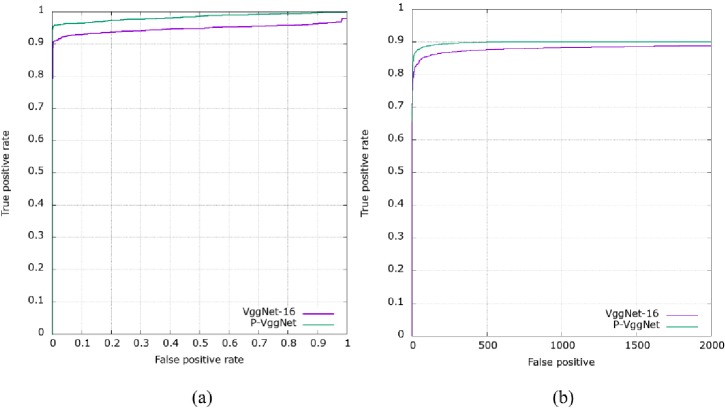
Results on the LWF dataset and FDDB dataset.

The two trained models were tested on the LWF dataset [27] and FDDB dataset [28]. Fig 9(A) shows the results on the LWF dataset. As seen in Fig 9(A), with the same false positive rate, the true positive rate of modeling with P_VggNet is always higher than that with VggNet-16. Fig 9(B) shows the result on the FDDB dataset. As seen in Fig 9(B), with the same false positive number, the true positive rate of modeling with P_VggNet is always higher than that with VggNet-16.

## 6 Conclusions

In this paper, we proposed a new CNN structure, P_VggNet. P_VggNet was VggNet-16 with P_Net_A and P_Net_B. The core concept of P_Net was changing the image pixels to weights according to the relationship between weight and pixel. To evaluate P_VggNet, we used VggNet-16 as the baseline approach, and two experiments were designed. Experiment 1 was designed to test their performance on image recognition. The results on the CIFAR-10 and CIFAR-100 datasets showed that the training time of P_VggNet was slightly longer than that of VggNet-16, but P_VggNet could achieve higher accuracy and less loss than VggNet-16 could, which means that P_VggNet is more efficient than VggNet. Experiment 2 was designed to test their performance on face detection. We changed the convolutional layers in ONet of MTCNN to P_VggNet and VggNet-16 and trained the two MTCNN models on the WIDER Face dataset and Celeba dataset. All results on the LWF dataset and FDDB dataset indicated that the true positive rate of modeling with P_VggNet is higher than that with VggNet-16. In summary, P_VggNet extracts image features more efficiently than VggNet-16 does.
